# Short-term infraorbital nerve electrical stimulation for herpes zoster herpetic trigeminal maxillary division neuralgia: A case report

**DOI:** 10.1097/MD.0000000000045720

**Published:** 2026-01-30

**Authors:** Meng Hu, Mei Rui Li, Jin Meng Song, Jin Hua Zhao, Guang Jian Zhang

**Affiliations:** a Department of Pain, Affiliated Hospital of Yanbian University, Yanji City, Jilin Province, China.

**Keywords:** herpes zoster neuralgia, infraorbital nerve, maxillary division, short duration electrical stimulation, trigeminal nerve

## Abstract

**Rationale:**

Herpetic neuralgia of the maxillary division of the trigeminal nerve is a rare clinical condition characterized by unilaterally distributed burning pain with clustered blister-like lesions. Peripheral nerve stimulation, an effective neuromodulation technique, has demonstrated significant efficacy in treating herpetic neuralgia. However, the application of peripheral nerve stimulation to herpes zoster (HZ) of the maxillary division of the trigeminal nerve is still limited. This study reports a case of HZ trigeminal neuralgia maxillary division neuralgia treated with a short-term infraorbital nerve stimulation guided by digital subtraction angiography (DSA) with satisfactory results.

**Patient concerns:**

The patient, a 77-year-old male, was admitted to the hospital with “left-sided facial pain with herpes for 1 month.” On examination, the left side of the face showed an incompletely dislodged crust and hyperpigmented scar, and the lesion was confined to the infraorbital innervation area. It did not cross the midline of the face, with a positive trigger pain test. Pain assessment: Numerical Rating Scale (NRS) 7 to 8, nocturnal eruptive pain episodes 3 to 4 times/night, Pittsburgh Sleep Quality Index 19 to 20.

**Diagnosis:**

Herpes zoster neuralgia of the maxillary division of the trigeminal nerve.

**Interventions:**

DSA-guided short-term infraorbital nerve electrical stimulation placement.

**Outcomes:**

After a short-term infraorbital nerve stimulation, the patient experienced significant relief of pain symptoms and complete healing of the skin lesions. The NRS score decreased from 7 to 8 before treatment to 2 to 3, and the nocturnal eruption pain disappeared, significantly improving sleep quality. Postoperative follow-up at 1 and 3 months showed that the NRS score decreased to 1 to 2, the Pittsburgh Sleep Quality Index showed a progressive decrease, and sleep quality improved.

**Lessons:**

Our findings suggest that DSA-guided short-term infraorbital nerve electrical stimulation offers a new option for treating pain in patients with HZ herpetic trigeminal maxillary division neuralgia when commonly used clinical treatments are ineffective.

## 1. Introduction

The reactivation of the varicella-zoster virus latent in the trigeminal ganglion triggers herpes zoster (HZ) trigeminal neuralgia. In cases of HZ of the head and face, the trigeminal nerve is involved in up to 58% of cases, with involvement of the maxillary nerve being the least common.^[1,2]^ The typical clinical features of this condition are unilaterally distributed burning pain with clustered blister-like lesions distributed along the course of the involved nerves. Epidemiologic data show that the global cumulative incidence of HZ ranges from 2.9 to 19.5/1000 person-years, with an annual incidence of 5.23 to 10.9/1000 person-years, and that the incidence tends to increase significantly with age. Notably, 5% to 30% of patients with HZ develop postherpetic neuralgia (PHN), which is the most common complication of HZ.^[3]^ Patients with PHN often present with severe cutting, burning, or electric shock-like pain, which is often accompanied by complications such as sleep disturbances and mood disorders.^[4]^ Currently, medication is still the 1st-line treatment option for PHN, but long-term use may lead to problems such as drug dependence and adverse reactions. Although infraorbital nerve block is widely used in treating HZ trigeminal neuralgia, some patients have problems such as short duration of efficacy and unsatisfactory therapeutic effect.^[5]^ Peripheral nerve stimulation (PNS) is an effective neuromodulation technique that reduces pain perception by modulating the electrical activity of peripheral nerves and altering the conduction of pain signals to the central nervous system. This technique has achieved significant clinical efficacy in treating HZ neuralgia.^[6]^ However, clinical reports of infraorbital nerve electrical stimulation for herpetic neuralgia of the maxillary division of the trigeminal nerve are still limited. In this study, we report a case of herpetic HZ neuralgia of the maxillary division of the trigeminal nerve treated with digital subtraction angiography (DSA)-guided electrostimulation of the infraorbital nerve with satisfactory efficacy.

## 2. Case report

A 77-year-old male presented to Yanbian University Hospital on October 20, 2023, with left-sided facial pain with herpes for 1 month. The patient had a history of type 2 diabetes mellitus, and 30 days ago, he developed left-sided facial pain with no apparent cause, which radiated along the maxillary nerve distribution area, with persistent pins and needles and burning pain, accompanied by autonomic symptoms such as tearing and runny nose, and apparent tenderness, and 4 days later, a clustered herpes-like rash appeared in the area of pain. He was admitted to the Department of Dermatology of the local hospital. During the treatment, oral pregabalin (150 mg/d), valaciclovir (1000 mg 3 times a day), methylcobalamin (0.5 mg 3 times a day), and topical 3% ganciclovir cream were administered. However, the pain relief was not noticeable. Two weeks later, all the rashes were crusted with hyperpigmentation, and he was referred to the Department of Pain Management because of the persistent pain.

**Figure 1.**
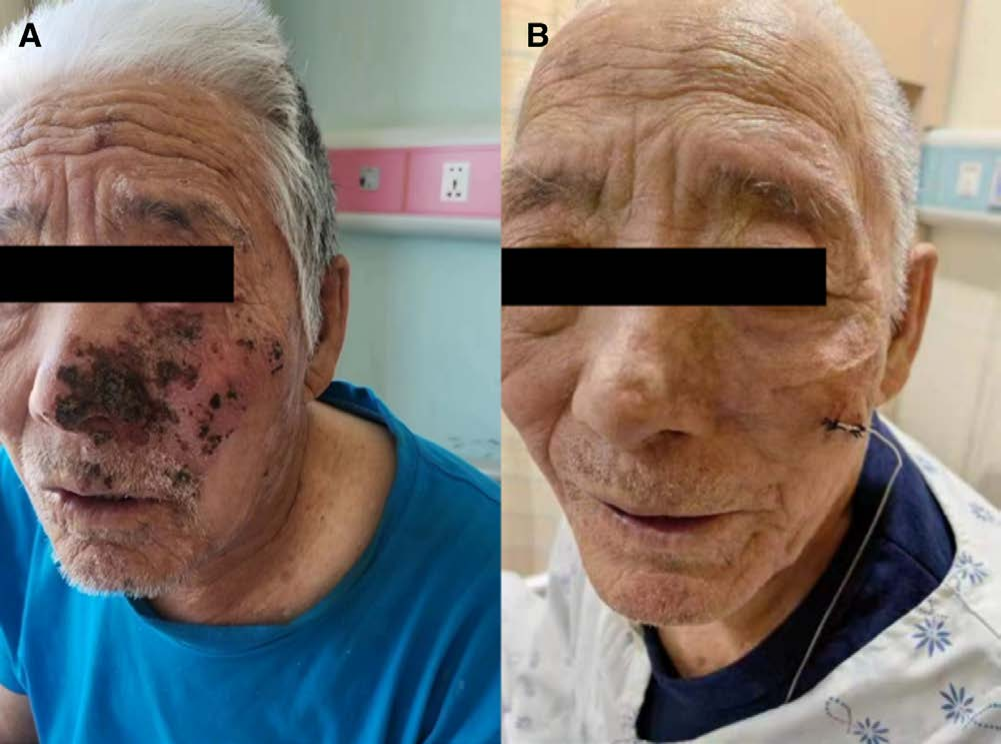
(A) Incompletely dislodged crust and hyperpigmentation are seen in the distribution area of the maxillary division on the left side. (B) The patient’s left side crust was completely removed at the time of discharge.

Physical examination: 12 pairs of cranial nerves were examined without apparent abnormalities; incompletely dislodged crusts and hyperpigmented scarring were seen on the left side of the face, and the lesion was confined to the infraorbital innervation area and did not cross the median line of the face (Fig. 1), with a positive trigger pain test. Pain assessment: Numeric Rating Scale (NRS) 7 to 8 points, nocturnal eruptive pain episodes 3 to 4 times/night, Pittsburgh Sleep Quality Index (PSQI) 19 to 20 points. Cranial CT examination suggested multiple lacunar cerebral infarcts, ischemic cerebral white matter lesions, and left thalamic calcified foci to be drained. Final diagnosis: HZ maxillary division of trigeminal nerve neuralgia.

After admission, a comprehensive treatment plan was given: antiviral therapy: pregabalin 150 mg every 12 hours; neurotrophic: methylcobalamin 0.5 mg 3 times daily; analgesic therapy: aminophenol oxycodone tablets (each tablet contains oxycodone hydrochloride 5 mg + acetaminophen 325 mg) taken every 6 hours. Under ultrasound guidance, the patient underwent continuous left infraorbital nerve block and left stellate ganglion block treatment for 4 days. Drug-related adverse reactions, including nausea, vomiting, dizziness, and constipation, occurred during treatment. Given the above, PNS treatment was proposed after the patient’s informed consent.

## 3. Treatment process

### 3.1. Neurological blockade process

The patient underwent 4 consecutive days of ultrasound-guided left infraorbital nerve and stellate ganglion block. The procedure, performed under standard monitoring, utilized the precision and safety of ultrasound. For the infraorbital nerve block, the ultrasound was used to locate the infraorbital foramen, followed by the injection of 3 mL of 0.3% ropivacaine. The stellate ganglion block involved injecting an equal volume of ropivacaine at the lower margin of the C6 transverse process. The patient’s successful response was indicated by the development of Horner syndrome, without any complications.

#### 3.1.1. Results

During the 4-day nerve block treatment, pain relief exhibited a time-dependent characteristic: the NRS score decreased to 3 to 4 points 1 hour post-block, increased to 5 to 6 points 6 hours post-block, and returned to pretreatment levels (6–7 points) by 24 hours post-block. Although this treatment effectively controlled acute pain, the analgesic effect was limited in duration (lasting < 24 hours) (Table 1) and breakthrough pain episodes occurred (2–3 times daily). Based on this, on the 5th day, an infraorbital nerve stimulator was implanted under DSA guidance to achieve long-term pain relief.

**Table 1:** Changes in NRS scores before and after obstruction.

NRS days	NRS before blockage	NRS 1 hour after blockage	NRS 6 hours after blockage	NRS 24 hours after blockage
1	7	3	6	6
2	6	4	6	7
3	6	3	6	7
4	7	4	5	7

NRS = numeric rating scale.

### 3.2. Surgical procedure

**Figure 2.**
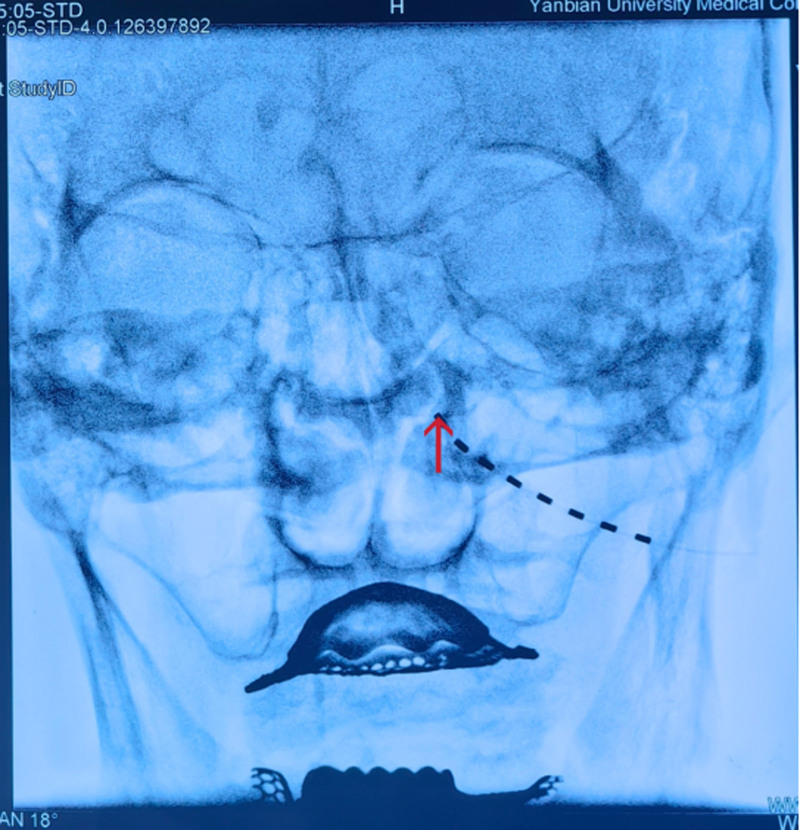
The arrow shows: the end of the electrode for electrical stimulation is placed on the left infraorbital nerve.

The patient was supine, intravenous access was established, and electrocardiogram, noninvasive blood pressure, and oxygen saturation were continuously monitored. The puncture point was positioned 1 cm below the infraorbital rim. After local infiltration anesthesia (1% lidocaine), the implantable spinal cord nerve stimulation electrode (model: L3213, Beijing Pinch Medical Equipment Co., Ltd.) was inserted into the bone surface along the medial-superior direction (infraorbital foramen) using an implanted puncture needle. After confirming that there was no blood or cerebrospinal fluid, the electrode was slowly inserted under DSA guidance so that the end of the electrode was positioned at the lateral edge of the middle turbinate. Subsequently, the puncture needle was withdrawn, the stimulator was connected, and the electrical stimulation parameters were set (voltage: 0.5 V, pulse width: 40 μs, and frequency: 60 Hz) to induce a tolerable soreness, numbness, and electrocution in the painful area. Finally, the electrodes were sutured and fixed, and the puncture point was covered with a sterile dressing and pressed for 5 minutes. Postoperative X-ray images confirmed the electrode position (Fig. 2).

#### 3.2.1. Observation indicators

NRS scores and PSQI scores were assessed on days 1, 3, 8, and 9 postoperatively, as well as at 1 month and 3 months postoperatively. Pregabalin dosage was recorded (Table 2).

**Table 2:** Patient preoperative data and outcome indicators.

Days	0	1	3	8	9	30	90
NRS	7	5	3	2	2	2	2
PSQI	19	16	11	6	5	4	4
Pregabalin dosage (mg)	150	150	150	150	150	0	0

NRS = numeric rating scale, PSQI = Pittsburgh Sleep Quality Index.

#### 3.2.2. Result and follow-up

Following the implantation of electrode leads during surgery, continuous single-pulse electrical stimulation therapy was administered (electrode retention time: 8 days). On the second postoperative day, the electrical stimulation parameters were adjusted to (voltage: 1.05 V, pulse width: 60 μs, frequency: 80 Hz), with further adjustments made based on the patient’s pain coverage area and tolerance. The final parameters were then applied continuously until the eighth postoperative day (Table 3). Under electrical stimulation therapy, pain was significantly alleviated, with the NRS score decreasing from 7 to 8 to 2 to 3 (Table 2), nocturnal flare-ups of pain disappeared, and sleep quality improved markedly. Pain did not recur 24 hours after the electrical stimulation was discontinued, and the therapeutic effect persisted. The electrode leads were removed, and no treatment-related complications occurred. The patient was discharged on the 9th day postoperatively. During hospitalization, the patient’s rash completely healed, and the scabs fell off (Fig. 1). Follow-up at 1 month and 3 months post-discharge showed that the NRS score further decreased to 1 to 2 points, and the PSQI score continued to decline progressively (Table 2).

**Table 3:** Changes in spinal cord electrical stimulation parameters.

Days	1	2	3	4	5	6	7	8
V	0.5	1.05	1.35	1.5	1.8	1.8	1.8	1.8
μs	40	60	70	90	90	90	90	90
Hz	60	80	90	120	120	120	120	120

Hz = frequency, V = voltage, μs = pulse width.

## 4. Discussion

This study reports a case of HZ herpeticus trigeminal maxillary division neuralgia that was successfully treated with PNS. The case had typical clinical manifestations: the pain was distributed along the maxillary division of the trigeminal nerve unilaterally and was strictly confined to 1 side of the body’s midline. The nature of the pain was persistent pinprick-like and burning pain, accompanied by autonomic symptoms such as tearing, runny nose and headache during the attack. The patient’s pain was only temporarily relieved after systemic medication (including antiviral, analgesic, and neurotrophic) combined with nerve block therapy. Short-term infraorbital nerve stimulation was performed under local anesthesia to cover the painful area by adjusting the stimulation parameters. After 8 days of continuous treatment, the patient’s pain was significantly relieved, the dose of analgesic drugs was gradually reduced, and no serious complications occurred.

HZ is a neurocutaneous disease caused by the reactivation of the latent varicella-zoster virus. The main risk factors for HZ include advanced age, human immunodeficiency virus infection, diabetes mellitus, malignancy, immunosuppressive therapy, and psychological stress.^[7]^ Two apparent risk factors were present in our patient: advanced age (77 years) and history of type 2 diabetes mellitus. Epidemiologic studies have shown that the lifetime risk of HZ in the general population is approximately 20% to 30%. The incidence increases significantly after the age of 50 years, with a cumulative incidence approaching 50% by the age of 85 years.^[8]^ The hyperglycemic state can lead to immune dysfunction and alterations in the ganglionic microenvironment, prompting reactivation of varicella-zoster virus latent in the trigeminal ganglion, which in turn leads to cutaneous herpes and nerve damage.^[9,10]^

Currently, medication is still the 1st-line treatment option for PHN. However, long-term use may lead to adverse effects such as sedation, dry mouth, blurred vision, weight gain, urinary retention, and peripheral edema, which affects patient compliance with treatment.^[11]^ The interventional treatment modalities for HZ neuralgia are diverse, including nerve block, transcutaneous electrical nerve stimulation, pulsed radiofrequency, spinal cord electrical stimulation, peripheral electrical nerve stimulation, dorsal root ganglion destruction, and implantation of intrathecal drug infusion system.^[12]^ Among them, the efficacy of nerve block is maintained for a relatively short period of time. Since Wall and Sweet 1st reported nerve electrical stimulation for analgesia in 1967, studies have shown that PNS’s long-term efficacy for treating PHN can reach 70% to 86.4%.^[13,14]^

As a terminal division of the maxillary nerve, the infraorbital nerve, after emanating from the trigeminal ganglion, passes through the lateral wall of the cavernous sinus, the round foramen to the pterygopalatine fossa, and then enters the orbit through the infraorbital fissure and finally exits through the infraorbital foramen to innervate sensation in the region of the lower eyelid, the nose, and the upper lip. Due to its superficial anatomical location, the infraorbital nerve is one of PNS’s most suitable trigeminal divisions.^[5]^ DSA-guided short-term infraorbital nerve electrical stimulation is easy to perform, time-consuming, and well-tolerated by patients. The electrodes were left in place for only 8 days, with no complications occurring during the treatment period, and the recent efficacy of the treatment was remarkable: NRS was significantly reduced, sleep quality improved, and pregabalin could be gradually discontinued. Follow-up at 3 months showed continued satisfactory pain control.

This study has the following limitations: first, as a single case report, the sample is not representative, and future studies should be designed as rigorous randomized controlled trials to validate the efficacy and safety of this treatment regimen. Second, the 3-month follow-up period may not be sufficient to assess the long-term effects of treatment and potential complications fully. Prolonging the follow-up period in subsequent studies would help obtain more reliable long-term prognosis data.

## 5. Conclusion

This case report demonstrates that DSA-guided short-term electrical stimulation of the infraorbital nerve provides significant pain relief and offers a new option for the clinical management of HZ herpetic trigeminal maxillary division neuralgia.

## Acknowledgments

I would like to thank all medical staff of the Department of Pain.

## Author contributions

**Conceptualization:** Meng Hu, Jin Meng Song, Jin Hua Zhao.

**Data curation:** Meng Hu, Jin Meng Song.

**Supervision:** Mei Rui Li, Guang Jian Zhang.

**Writing – original draft:** Meng Hu.

**Writing – review & editing:** Meng Hu, Mei Rui Li, Guang Jian Zhang.
